# Activities of daily living associated with postoperative intensive care unit survival in elderly patients following elective major abdominal surgery: An observational cohort study

**DOI:** 10.1097/MD.0000000000026056

**Published:** 2021-06-04

**Authors:** Yu Kang, Gui-Chen Zhang, Ji-Qiao Zhu, Xiang-Yang Fang, Jing Niu, Ying Zhang, Xiao-Juan Wang

**Affiliations:** a Department of Geriatric Medicine; b Department of Surgical Intensive Care Unit; c Department of Liver and Gallbladder Surgery, Beijing Chaoyang Hospital, Capital Medical University, Beijing, China.

**Keywords:** activities of daily living, elderly, major abdominal surgery, intensive care unit survival, preoperative assessment

## Abstract

Elderly patients who undergo major abdominal surgery are being in increasing numbers. Intensive care unit (ICU) survival is critical for surgical decision-making process. Activities of daily living (ADL) are associated with clinical outcomes in the elderly. We aimed to investigate the relationship between ADL and postoperative ICU survival in elderly patients following elective major abdominal surgery.

We conducted a retrospective cohort study involving patients aged ≥65 years admitted to the surgical intensive care unit (SICU) following elective major abdominal surgery. Data from all patients were extracted from the electronic medical records. The Barthel Index (BI) was used to assess the level of dependency in ADL at the time of hospital admission.

ICU survivors group had higher Barthel Index (BI) scores than non-survivors group (*P* < .001). With the increase of BI score, postoperative ICU survival rate gradually increased. The ICU survivals in patients with BI 0–20, BI 21–40, BI 41–60, BI 61–80 and BI 81–100 were 55.7%, 67.6%, 72.4%, 83.3% and 84.2%, respectively. In logistic regression, The Barthel Index (BI) was significantly correlated with the postoperative ICU survival in elderly patients following elective major abdominal surgery (OR = 1.33, 95% CI: 1.20–1.47, *P* = .02). The area under the receiver operating characteristic (ROC) curve of Barthel Index in predicting postoperative ICU survival was 0.704 (95% CI, 0.638–0.771). Kaplan–Meier survival curve in BI≥30 patients and BI < 30 patients showed significantly different.

Activity of daily living upon admission was associated with postoperative intensive care unit survival in elderly patients following elective major abdominal surgery. The Barthel Index(BI) ≥30 was associated with increased postoperative ICU survival. For the elderly with better functional status, they could be given more surgery opportunities. For those elderly patients BI < 30, these results provide useful information for clinicians, patients and their families to make palliative care decisions.

## 1 Introduction

The world's population is aging, which subsequently leads to an increase in elderly patients who undergo major abdominal surgery.^[1]^ As the life expectancy increases, patients older than 65 years account for a large fraction of surgeries. Predictions that the aging of the population will cause further growth in the utilization of surgical services.^[2]^ Surgical decision making in elderly patients can be particularly challenging. Older people have a higher risk of surgery.^[3]^ Although innovations in perioperative management have led to a rise in surgical procedures performed in older patients with complex medical needs, many of elderly patients were managed non-operatively. However, in many cases, patients with surgery had a satisfactory outcome in contrast to those without surgery.^[4–6]^ Despite higher risk among elderly patients, major surgery can be performed safely in the very old.^[7–8]^ Age no longer clear contraindications to surgery. Preoperative assessment in the elderly population requires a holistic approach.

There is growing evidence that the functional status, is more important than age and comorbidity in predicting prognosis in the elderly.^[9]^ Functional status decline is increasing with aging.^[10,11]^ About a quarter of adults reported moderate or severely limited functioning between ages 45 to 65 years, and nearly half of those older than 65 years reported these limitations.^[12]^ Activities of daily living (ADL) assessment is a convenient way to assess a person's functional level. Even small changes in the ADL functional level are associated with clinically relevant outcomes. ADL are associated with clinical outcomes in acute medical patients,^[13]^ geriatric trauma,^[14]^ hip fractures,^[15–16]^ pulmonary infections,^[17]^ and heart failure^[18]^. ADL evaluation of the elderly could be conducive to clinical decision-making process.

As the increase in the number of elderly patients who undergo surgery, patients admitted to intensive care unit (ICU) following major abdominal surgery are increasing.^[19–21]^ ICU medicine frequently determines the survival or death after surgery. As ICU survival following surgery is such an important subject for the surgery in the elderly, postoperative ICU survival is critical for surgical decision-making process. However, no data about relationship between ADL and postoperative ICU survival in elderly patients following elective major abdominal surgery are available to date. A better understanding of these outcomes would benefit patients and clinicians in setting appropriate expectations before surgery and optimizing perioperative management. Thus, here we aimed to determine whether activity of daily living (ADL) are associated with postoperative ICU survival in elderly patients following elective major abdominal surgery.

## 2 Methods

### 2.1 Study subjects

We conducted a retrospective cohort study involving patients aged ≥65 years admitted to the surgical intensive care unit (SICU) following elective major abdominal surgery in Beijing Chao-yang Hospital, Capital Medical University, between June 2012 and June 2020. Beijing Chao-Yang Hospital has 1,900 beds including 20 beds in SICU.

### 2.2 Data collection

Data from all patients were collected from the electronic medical records. The following variables were collected: age, sex, the Barthel Index, surgical disease classification, the American society of anesthesiologists (ASA) physical status class, estimated blood loss, operative duration, co-morbidity, vital signs, length of ICU stay and vital status. All patient was transferred to the SICU after surgery. If a patient was admitted to the ICU several times, this was considered as multiple stays and data were analyzed for first stay after surgery. Survival status or date of death were collected for all patients during the ICU stay. The study protocol was approved by the Institutional Review Board for Human Studies of Beijing Chaoyang Hospital, Beijing, China. The informed consent were exempted because this was a retrospective study. Patients’ data confidentiality was fully respected during data collection and the preparation of the manuscript.

### 2.3 ADL evaluation

All patients underwent ADL evaluation by primary duty nurse within 2 h of admission to general surgery or hepatobiliary surgery ward. The Barthel Index (BI) was used to assess the level of dependency in ADL at the time of hospital admission. The BI measures ten functions that are important for independent living^[22]^: feeding, dressing, transferring, grooming, bathing, toileting, walking, stair climbing, bowel control, and bladder care. BI score ranging from 0 to100 points, higher BI score indicates lower dependency (Table S1 Supplemental Digital Content ).

### 2.4 Statistical analysis

Continuous variables were expressed as the mean and standard deviation, or median and interquartile range for non-normally distributed data. Significant differences between two groups were determined by Student's *t*-test or Mann–Whitney *U* test for the continuous variables. Categorical variables were described using counts and percentages. Categorical data were tested with χ^2^ and Fisher's exact test. To determine the factors associated with the ICU survival logistic regression analysis was performed. The odds ratios (OR) with 95% confidence intervals (CI) were presented. The receiver operating characteristics (ROC) curves and the areas under the curves (AUCs) were applied in the model to assess the prognostic value. Survival rate was calculated using the Kaplan–Meier (KM) method. The statistical analyses of data were performed by using SPSS 20.0 (SPSS Inc., Chicago, IL, USA) and R software (version 3.3.2) with the corresponding R packages. All tests were two-sided, and a value of *P* < .05 was considered statistically significant.

## 3 Results

### 3.1 Comparison of the characteristics between ICU survivors and non-survivors

A total of 264 patients age≥65 years admitted to the surgical intensive care unit (SICU) following elective major abdominal surgery were included in the study. Group analysis was performed between ICU survivors (n = 178) and non-survivors (n = 86). The characteristics of the 264 patients in the study are shown in Table 1.

**Table 1:** Characteristics of postoperative ICU survivors and non-survivors in elderly patients following elective major abdominal surgery.

Characteristic	ICU survivors n = 178	ICU Non-survivors n = 86	*P* value^∗^
Age, years	73.9 ± 7.2	76.5 ± 7.4	.985
Male, n (%)	105 (58.9)	55 (63.9)	.502
Barthel index (BI)	42.3 ± 31.6	24.1 ± 26.5	<.001
Disease classification, n (%)			.192
Esophagogastric,	37 (20.8)	19 (22.1)	
Hepatic	12 (6.7)	5 (5.8)	
Pancreaticobiliary	68 (38.2)	21 (24.4)	
Colorectal	33 (18.5)	22 (25.6)	
Other	28 (15.7)	19 (22.1)	
ASA class ≥3, n (%)	81 (45.5)	50 (58.1)	.036
Estimated blood loss, mL	190.2 ± 232.2	589.6 ± 1096.1	.006
Operative duration ≥3 hours	29 (16.3)	41 (47.7)	<.001
Comorbidities, n (%)			
Smoking	121 (67.9)	52 (60.5)	.564
Coronary artery disease	29 (16.3)	19 (22.1)	.307
Diabetes	33 (18.5)	17 (19.8)	.867
Hypertension	89 (50.0)	33 (38.4)	.087
Chronic renal failure	31 (17.4)	29 (33.7)	.003
Vital signs			
Respiratory rate (per minute)	22.8 ± 9.8	23.5 ± 7.9	.618
Heart rate (bpm)	95.4 ± 19.1	98.9 ± 23.1	.334
SBP (mmHg)	128.4 ± 25.9	119.6 ± 27.7	.012
Body temperature (°C)	36.9 ± 0.8	37.1 ± 0.9	.367
Impaired consciousness, n (%)	15 (8.4)	26 (30.2)	<.001
Length of ICU stay	24.6 ± 10.3	16.6 ± 6.7	<.001

Mean BI score in the ICU survivors group and non-survivors group were 42.3 ± 31.6 and 24.1 ± 26.5, respectively. ICU survivors group had higher BI scores than non-survivors group (*P* < .001). There were no significant differences in age and sex. The ratio of patients ASA class ≥3 or operative duration≥3 h were significantly different between the two groups (*P* = .036, *P* < .001, respectively). While, estimated blood loss during the operation were different between the two groups (*P* = .006). As for the distribution of disease classification, there was not a significantly difference between the two groups (*P* = .192). The non-survivors group patients had a higher percentage of chronic renal failure history (*P* = .003) and impaired consciousness (*P* < .001).

### 3.2 Association between ADL and postoperative ICU survival

**Figure 1:**
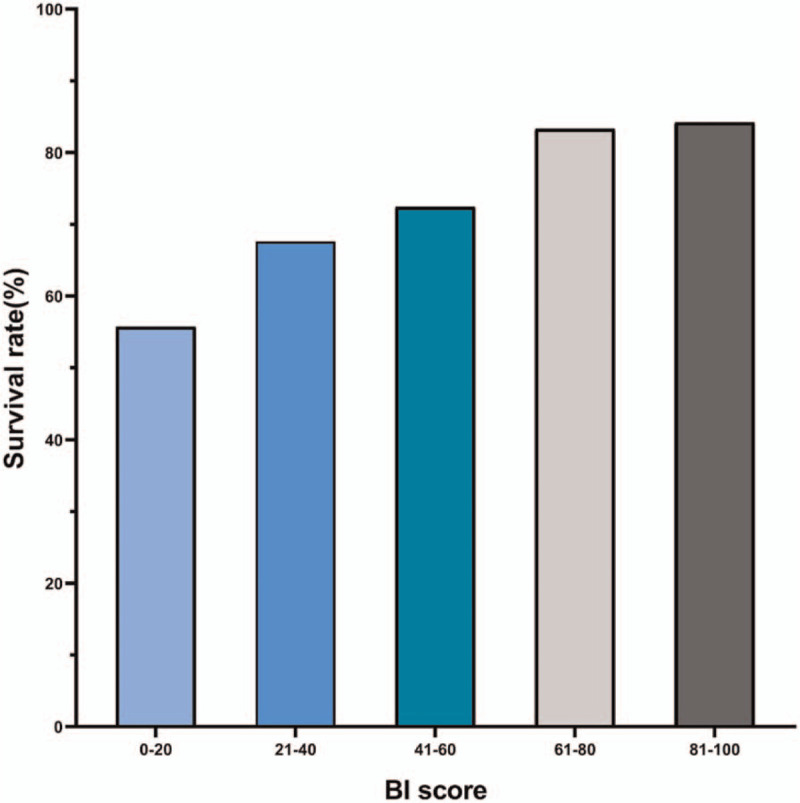
Intensive care unit survival rates of elderly patients following elective major abdominal surgery according to the Barthel Index. BI = Barthel Index

As shown in Figure 1, a higher BI was associated with increased postoperative ICU survival in elderly patients following elective major abdominal surgery. In all 264 patients, the ICU survival in patient with BI 0–20, BI 21–40, BI 41–60, BI 61–80 and BI 81–100 were 55.7%, 67.6%, 72.4%, 83.3% and 84.2%, respectively.

We also investigated the association between ADL at admission and postoperative ICU survival using logistic regression. In logistic regression model (Table 2), The Barthel Index (BI) was significantly correlated with the postoperative ICU survival in elderly patients following elective major abdominal surgery (OR = 1.33, 95% CI: 1.20–1.47, *P* = .02).

**Table 2:** Logistic regression analyses of factors associated with the postoperative ICU survival in elderly patients following elective major abdominal surgery^∗^.

Characteristics	OR (95% CI)	*P* value
ADL (BI score)	1.33 (1.20–1.47)	.02
Operative duration ≥3 hours	0.66 (0.62–0.74)	<.01
ASA class ≥3	0.72 (0.64–0.80)	.02

### 3.3 ADL and prediction of postoperative ICU survival

**Figure 2:**
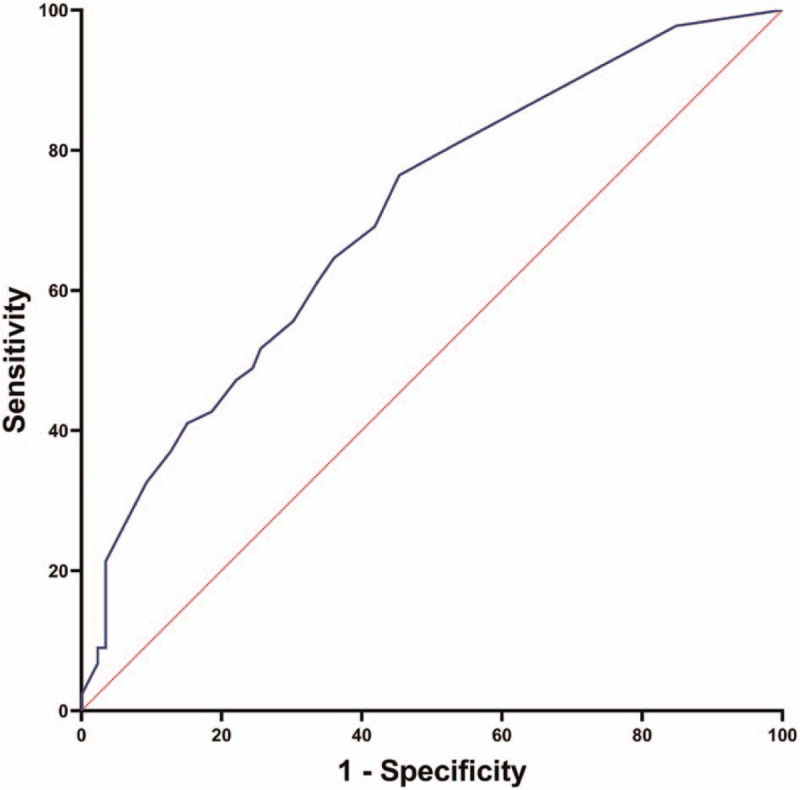
The receiver operating characteristic curve of Barthel Index in predicting Intensive care unit survival in elderly patients following elective major abdominal surgery.

We examined the role of Barthel Index as a predictor of postoperative ICU survival in elderly patients following elective major abdominal surgery. The receiver operating characteristic (ROC) curve for the postoperative ICU survival based on BI score is presented in Figure 2. The area under the ROC curve of Barthel Index in predicting postoperative ICU survival was 0.704 (95% CI, 0.638–0.771). Using Youden index, the best cut-off point of BI score was 28.5 for ICU survival in elderly patients following elective major abdominal surgery.

**Figure 3:**
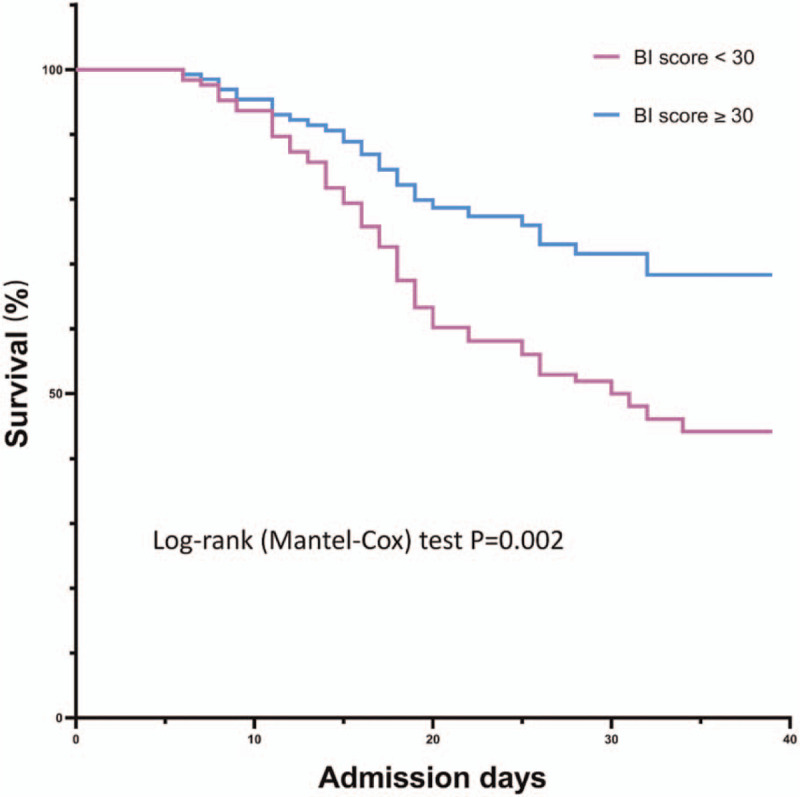
Kaplan–Meier curve of intensive care unit survival in elderly patients following elective major abdominal surgery according to the Barthel Index (BI≥30 or < 30). BI = Barthel Index.

Kaplan–Meier curves of postoperative ICU survival in elderly patients following elective major abdominal surgery according to the BI score are showed in Figure 3. Kaplan–Meier survival curve in BI≥30 patients and BI < 30 patients showed significantly different.

## 4 Discussion

The main finding in this study was that better ADL status at admission was associated with increased postoperative ICU survival in elderly patients following elective major abdominal surgery. We noted that BI score at admission could be a predictor of postoperative ICU survival. Patients with a higher BI showed better clinical outcome. For the elderly with good functional status at admission, they could be given more surgery opportunities.

Surgical decision making in elderly patients can be particularly challenging. Although innovations in perioperative management have led to a rise in surgical procedures performed in older patients with complex medical needs, many of elderly patients were managed non-operatively,^[23]^ majority of them due to questionable clinical outcome. As a matter of fact, patients with surgery had a satisfactory outcome in contrast to those without surgery in many cases,^[3–5]^ major surgery can be performed safely in the very old.^[7–8]^ Thus, preoperative assessment in the elderly population requires a holistic approach. There is growing evidence that the functional status, is more important than age and comorbidity in predicting prognosis in the elderly.^[9]^ ADL is a convenient way to assess a person's functional level, even small changes in the ADL functional level are associated with clinically relevant outcomes.^[14–18]^ It has been reported that dependence in activities of daily living was one of the predictors of in-hospital mortality in older general surgical patients.^[24]^ Dependency in ADL have been proved to influence survival in older patients attending the emergency department or being hospital with orthopaedic or trauma conditions.^[25–29]^ Frailty and delay in management are associated with poor surgical outcomes after emergency abdominal surgery in the elderly.^[30]^

Previous studies have shown that functional status in elderly patients are frequently individualized. Increasing age does not always mean poor functional status. ADL functional status could be more important than age in surgical decision making process in the elderly. The importance of preoperative functional evaluation in the elderly has been gradually acknowledged among more surgical specialties over time. Hitherto, association between ADL function at admission and postoperative ICU survival in elderly patients following elective major abdominal surgery has not been studied.

ADL reflects the functional status and self-care ability of the elderly. Barthel Index is a widely used functional assessment of ADL. The Barthel Index (BI) is the official ADL tool of geriatric patients. All patients admitted to ward were evaluated in our hospital. BI is reliable, simple, and it can be used as a conventional method for the assessment of the ADL functional status at admission in geriatric patients. ADL should be included in preoperative evaluation and that is conducive for surgical decisions making.

Another commonly used ADL scale is the Katz Index (KI). Along with the Barthel Index, it has been widely used for ADL evaluation of patients regardless of their medical condition. The Katz Index evaluates ability to perform six ADL. The Barthel Index and the Katz Index have previously been compared in elderly patients.^[31,32]^ Previous study showed that the Barthel Index might be a better scale than the Katz Index for the assessment of functional status of patients discharged from ICU, since it presented better discrimination of the ability to carry out the tasks.^[33]^

Intensive care units are confronted with increasing demand for the elderly, elderly patients now representing up to 20–30% of all ICU admissions.^[34–35]^ With the increase in the number of elderly patients who undergo surgery, patients admitted to ICU following major abdominal surgery are being in increasing numbers.^[19–21]^ Identifying potential factors for ICU survival in elderly patients and predicting the clinical outcome is crucial in surgical decision making. The postoperative ICU survival rate observed in this study was 67.4%. Studies focused on elderly patients admitted to ICU after operations are scarce. Anna et al retrospectively analyzed elderly patients who underwent emergency abdominal surgery and found that 81.9% survived to discharge and 87.6% survived for 30 days.^[35]^ The study by Monica Escher et al showed that critically ill elderly patients ICU and hospital mortality rates were 35.7% and 42.6% respectively.^[36]^ Monica et al reported that patients with median age 67 years admitted to the ICU, 71.1% survived for 28 days.^[37]^ Jennifer et al^[38]^ have reported the overall in-hospital mortality for nonagenarians who undergo abdominal operations is 15.2%. Our results were consistent with previous literature.

Different evaluation scales have been documented to assess in surgical risk, the most commonly be used are the ASA class. The ASA score was the most predictive factor analyzed, with a clear trend to increasing mortality with a higher score. However, ASA class is probably not an appropriate tool in the elderly cohort. ASA grading has been shown to have poor discriminatory performance when used as a comparator for a colorectal surgical risk score in a validation sample of 300 patients aged over 80 requiring emergency colectomy.^[39]^ Other factors that may influence the surgical outcome include the operative duration, estimated blood loss and comorbidities. However, in contrast to our study, data on ADL functional status was lacking. Previous study has shown that chronological age was not an independent factor of ICU mortality.^[40]^ Difference in clinical outcome is more likely dependent on functional status. A better understanding of the association of functional status on postoperative ICU survival would benefit patients and clinicians in setting appropriate expectations before surgery and optimizing perioperative management.

The study has some limitations. The first is the retrospective design of the study which resulted in some variables cannot be extracted from the electronic medical records. Secondly, our analysis was restricted to patients who underwent elective major abdominal surgery, we do not have information on patients with surgical problems who were not offered surgery. Thirdly, BI assessment has some limitations that may be influenced by the environment.

## 5 Conclusions

Activity of daily living upon admission was associated with postoperative intensive care unit survival in elderly patients following elective major abdominal surgery. The Barthel Index(BI) ≥30 was associated with increased postoperative ICU survival. For the elderly with better functional status, they could be given more surgery opportunities. For those elderly patients BI < 30, these results may provide useful information for clinicians, patients and their families to make palliative care decisions.

## Acknowledgments

The authors thank the patients, their families, and all investigators who participated in the study.

## Author contributions

**Investigation**: Yu Kang, Gui-Chen Zhang, Ji-Qiao Zhu, Xiang-Yang Fang, Jing Niu, Ying Zhang.

**Methodology**: Yu Kang, Gui-Chen Zhang, Ji-Qiao Zhu, Xiang-Yang Fang, Xiao-Juan Wang.

**Project administration**: Xiao-Juan Wang.

**Resources**: Xiang-Yang Fang, Xiao-Juan Wang.

**Software**: Yu Kang, Gui-Chen Zhang, Ji-Qiao Zhu.

**Validation**: Xiang-Yang Fang, Xiao-Juan Wang.

**Visualization**: Gui-Chen Zhang. Writing–original draft: Yu Kang.

**Writing – review & editing**: Yu Kang, Xiang-Yang Fang, Xiao-Juan Wang.

## Supplementary Material

Supplemental Digital Content
