# Supplementary material for: Activities of daily living associated with postoperative intensive care unit survival in elderly patients following elective major abdominal surgery: An observational cohort study
Source: Medicine (Baltimore). 2021 Jun 4;100(22):e26056. doi: 10.1097/MD.0000000000026056 (PMC8183836; doi:10.1097/MD.0000000000026056)
Supplement: Supplemental Digital Content [file medi-100-e26056-s001.docx]

**Supplementary Table 1. The Barthel Index** **scale**

| **The Barthel Index** | |
| --- | --- |
| **Bowels**  0=incontinent (or needs to be given enemata)  5=occasional accident(once/week)  10=continent | Patient's Score: |
| **Bladder**  0=incontinent, or catheterized and unable to manage  5=occasional accident(max. once per 24 hours)  10=continent(for over 7 days) | Patient's Score: |
| **Toilet use**  0=dependent  5=needs some help, but can do something alone  10=independent (on and off, dressing, wiping) | Patient's Score: |
| **Transfer**  0=unable-no sitting balance  5=major help (one or two people,physical),can sit  10= minor help (verbal or physical)  15= independent | Patient's Score: |
| **Mobility**  0=immobile  5=wheelchair independent, including corners, etc.  10=walks with help of one person (verbal or physical)  15= independent (but may use any aid, e.g., stick) | Patient's Score: |
| **Stairs**  0=unable  5=needs help (verbal, physical, carrying aid)  10= independent up and down | Patient's Score: |
| **Feeding**  0=unable  5=needs help cutting, spreading butter, etc.  10=independent (food provided within reach) | Patient's Score: |
| **Grooming**  0=needs help with personal care  5=independent face/hair/teeth/shaving (implements provided) | Patient's Score: |
| **Dressing**  0=dependent  5=needs help, but can do about half unaided  10= independent (including buttons, zips, laces, etc.) | Patient's Score: |
| **Bathing**  0=dependent  5=independent (or in shower) | Patient's Score: |
|  | **Total Score:** |
| Sum the patient's scores for each item. Total possible scores range from 0-100. | |
